# Thrombospondin-2 as a Predictive Biomarker for Hepatocellular Carcinoma after Hepatitis C Virus Elimination by Direct-Acting Antiviral

**DOI:** 10.3390/cancers15020463

**Published:** 2023-01-11

**Authors:** Takayuki Matsumae, Takahiro Kodama, Yuki Tahata, Yuta Myojin, Akira Doi, Akira Nishio, Ryoko Yamada, Yasutoshi Nozaki, Masahide Oshita, Naoki Hiramatsu, Naoki Morishita, Kazuyoshi Ohkawa, Taizo Hijioka, Mitsuru Sakakibara, Yoshinori Doi, Naruyasu Kakita, Takayuki Yakushijin, Ryotaro Sakamori, Hayato Hikita, Tomohide Tatsumi, Tetsuo Takehara

**Affiliations:** 1 Department of Gastroenterology and Hepatology, Osaka University Graduate School of Medicine, Suita 565-0871, Japan; 2 Center for Cancer Research, National Cancer Institute, Bethesda, MD 20892, USA; 3 Department of Gastroenterology and Hepatology, Kansai Rosai Hospital, Amagasaki 660-8511, Japan; 4 Department of Gastroenterology and Hepatology, Ikeda Municipal Hospital, Ikeda 563-0025, Japan; 5 Department of Gastroenterology and Hepatology, Osaka Rosai Hospital, Sakai 591-8501, Japan; 6 Department of Gastroenterology and Hepatology, Minoh City Hospital, Minoh 562-8562, Japan; 7 Department of Hepatobiliary and Pancreatic Oncology, Osaka International Cancer Institute, Osaka 541-8567, Japan; 8 Department of Gastroenterology and Hepatology, National Hospital Organization Osaka Minami Medical Center, Kawachinagano 586-8521, Japan; 9 Department of Gastroenterology and Hepatology, Yao Municipal Hospital, Yao 581-0069, Japan; 10 Department of Gastroenterology and Hepatology, Otemae Hospital, Osaka 540-0008, Japan; 11 Department of Gastroenterology and Hepatology, Kaizuka City Hospital, Kaizuka 597-0015, Japan; 12 Department of Gastroenterology and Hepatology, Osaka General Medical Center, Osaka 558-8558, Japan; 13 Department of Gastroenterology and Hepatology, National Hospital Organization Osaka National Hospital, Osaka 540-0006, Japan

**Keywords:** TSP-2, HCC, HCV, DAA, THBS2

## Abstract

**Simple Summary:**

Secreted glycoprotein thrombospondin-2 (TSP-2) is a predictive biomarker of hepatocellular carcinoma (HCC) occurrence in chronic hepatitis C (CHC) patients after HCV elimination by direct-acting antiviral agents (DAAs). The AFT score using TSP-2, AFP, and the FIB-4 index may identify those who require HCC surveillance.

**Abstract:**

We evaluated the value of secreted glycoprotein thrombospondin-2 (TSP-2) to predict hepatocellular carcinoma (HCC) occurrence in chronic hepatitis C (CHC) patients after Hepatitis C virus (HCV) elimination by direct-acting antiviral agents (DAAs). A total of 786 CHC patients without an HCC history who achieved a sustained virological response (SVR) with DAAs were randomly assigned 2:1, with 524 patients as the derivation cohort and 262 patients as the validation cohort. Serum TSP-2 levels at the end of treatment were measured by enzyme-linked immunosorbent assay (ELISA). In the derivation cohort, the cumulative HCC rate was significantly higher in the high TSP-2 group than in the low TSP-2 group. Multivariate Cox proportional hazards analysis revealed that TSP-2, α-fetoprotein (AFP), and the fibrosis-4 (FIB-4) index were independent HCC risk factors. The area under the receiver operating characteristic curve (AUROC) of the score calculated from these three factors (AFT score) for predicting HCC was 0.83, which was significantly higher than that of each factor alone (TSP-2: 0.70, AFP: 0.72, FIB-4: 0.69). The AFT score was used to stratify patients according to the risk of HCC occurrence in the validation cohort. Lastly, in patients with a FIB-4 index < 3.25, the serum TSP-2 levels could be used to identify those patients with a high risk of HCC occurrence. Serum TSP-2 levels are a predictive biomarker of HCC occurrence in CHC patients after HCV elimination by DAA treatment. The AFT score using TSP-2, AFP, and the FIB-4 index may identify those who require HCC surveillance.

## 1. Introduction

Direct-acting antiviral agents (DAAs) have become a standard treatment for hepatitis C virus (HCV) infection and can eradicate HCV with a more than 95% success rate regardless of the HCV genotype or host liver fibrosis status. HCV eradication is known to improve liver function and reduce the risk of hepatocellular carcinoma (HCC) occurrence [1,2] but cannot completely eliminate the risk of HCC development [3]. The molecular mechanisms of HCC development in DAA-cured sustained virological response (SVR) patients are not fully understood. However, the accumulation of genomic abnormalities, liver fibrosis, and cellular dysfunction caused by long-term persistent HCV infection remain after HCV elimination and may contribute to HCC development [4]. Comorbidities such as diabetes mellitus (DM) and concomitant liver injury caused by excess alcohol intake or nonalcoholic steatohepatitis (NASH) may also promote hepatocarcinogenesis [5]. Therefore, it is important to identify patients who have a high risk of HCC occurrence even after achievement of SVR by DAA treatment and to perform appropriate HCC surveillance [6]. 

It is frequently reported that patients with liver cirrhosis (F4) are at high risk of developing HCC [7,8], and the guidelines of the European Association for the Study of the Liver (EASL) and the American Association for the Study of Liver Disease (AASLD) recommend surveillance after viral eradication in patients with a fibrosis-4 (FIB-4) index > 3.25 who are considered to have cirrhosis [9]. However, although less frequent than in patients with a FIB-4 index > 3.25, HCC also occurs in patients with a FIB-4 index < 3.25 [7]. Indeed, the EASL recommends HCC surveillance for patients with advanced fibrosis (F3), but no circulating biomarkers have been proposed to identify those patients [10]. Therefore, it is desirable to develop better predictive biomarkers that can identify SVR patients with a high risk for HCC occurrence among noncirrhotic patients.

TSP-2, a member of the thrombospondin family, is a matricellular glycoprotein that mediates cell-to-cell and cell-to-matrix interactions [2]. We have recently reported that serum TSP-2 is a novel biomarker for identifying NASH, advanced fibrosis, and HCC occurrence among nonalcoholic fatty liver disease (NAFLD) patients [11]. In the present study, we examined the utility of serum TSP-2 as a biomarker of HCC occurrence in CHC patients who achieved SVR by DAA treatment.

## 2. Materials and Methods

### 2.1. Study Population

We conducted a prospective multicenter cohort study that enrolled 2840 CHC patients who underwent DAA treatment from September 2014 to December 2017 at Osaka University Hospital and 13 related hospitals. We excluded patients who were coinfected with human immunodeficiency virus or hepatitis B virus, suffered from primary bile cholangitis or autoimmune hepatitis, or were under 20 years old, or had undergone liver transplantation from the registration. Written informed consents were obtained from all patients, and the protocol of this study was reviewed and approved by the institutional review board (IRB) committees of Osaka University Hospital and all participating hospitals. IRB No of this study is 17032. This study complies with the Helsinki Declaration.

### 2.2. Regimens of DAA Treatment

The DAA treatment regimens were as follows: 24 weeks of daclatasvir and asunaprevir (DCV/ASV); 12 weeks of ombitasvir, paritaprevir and ritonavir (OBV/PTV/ritonavir), ledipasvir and sofosbuvir (LDV/SOF), elbasvir and grazoprevir (EBR/GZR), and SOF and ribavirin (SOF/RBV). We defined SVR as an undetectable serum HCV ribonucleic acid (RNA) level at 24 weeks after the end of treatment. All patients were treated according to the Japanese guidelines for the treatment of chronic HCV infection.

### 2.3. Patient Follow-Up

As surveillance for HCC, all patients in this study underwent abdominal ultrasonography, CT scan, or magnetic resonance imaging (MRI) every six months after the end of treatment (EOT) according to the recommendation of the Japan Society of Hepatology. The laboratory data including standard markers measured in clinical practice of chronic liver disease were collected in this study at the point of EOT (the HCV-RNA level was only measured at the pretreatment). The observation period started on the day that DAA treatment ended. The end point of the observation was the date when HCC occurred. In patients who never developed HCC, the date of the most recent liver imaging test was the last date of the observation period.

### 2.4. Enzyme-Linked Immunosorbent Assay (ELISA) of Serum TSP-2

The sera of patients registered in this study were stored in a −80 °C freezer at Osaka University. The serum TSP-2 level was examined with an ELISA kit for human TSP-2 (Catalog#DTSP20, R&D Systems, Minneapolis, MN, USA) according to the manufacturer’s instruction. The absorbance was examined by a Varioskan LUX (Thermo Scientific, Waltham, MA, USA).

### 2.5. Statistical Analysis

Statistical analysis was performed with Student’s t-test for comparing parametric values or the Mann–Whitney U test when comparing nonparametric values. For multiple comparisons, One-way ANOVA, followed by Tukey’s multiple comparisons test, was performed. The Kaplan–Meier curves were drawn and a log-rank test was performed to examine differences in the cumulative HCC occurrence rates. Kaplan–Meier curves were constructed using the period from the EOT date to the HCC occurrence date or the period from the EOT date to the death date or the last date of HCC surveillance. To examine the factors associated with HCC occurrence, univariate and multivariate Cox proportional hazards models were used. We used GraphPad Prism ver. 9.2.0 for Macintosh for data analysis and creating graphs.

## 3. Results

### 3.1. Serum TSP-2 Levels Are Capable of Identifying CHC Patients Who Have a High Risk of HCC Development after HCV Elimination by DAA Treatment

To evaluate the value of serum TSP-2 levels for predicting HCC occurrence in CHC patients who achieved SVR with DAAs, we selected 786 patients who fulfilled the following criteria from our multicenter prospective observational cohort: those with SVR achievement, those with complete medical records, those without a past HCC history, and those who provided agreement for serum storage (Supplementary Figure S1). The patients were randomly assigned 2:1, with 524 patients as the derivation cohort and 262 patients as the validation cohort. In the derivation cohort, the median age was 70 years, and the percentage of males was 38.9% (Table 1). The percentage of genotype 1 was 77.5%, and the median platelet count was 16.8 × 10^4^/μL (Table 1). 

**Figure 1:**
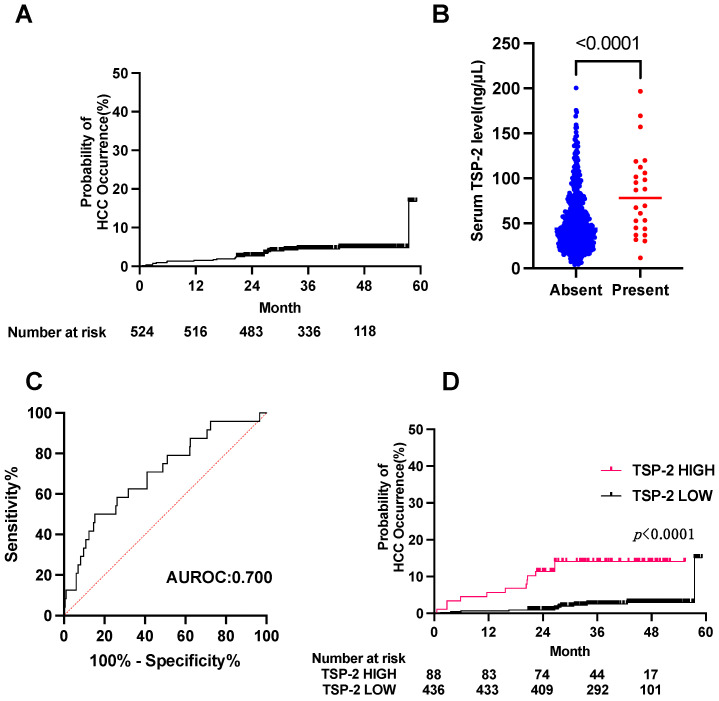
Serum TSP-2 levels can be used to identify CHC patients who have a high risk of HCC development after HCV elimination by DAA treatment. (**A**) Kaplan–Meier curve for HCC occurrence in the derivation cohort. (**B**) Serum TSP-2 levels in the HCC+ group and HCC− group in the derivation cohort. (**C**) ROC curve of TSP-2 for HCC occurrence in the derivation cohort. (**D**) Kaplan–Meier curves for the occurrence of HCC in patients in the derivation cohort divided by the TSP-2 Youden index level of 86.954 ng/µL.

A total of 24 patients (4.6%) developed HCC during the 41.5-month median observation period, and the cumulative HCC rate after DAA treatment was 2.7% at 2 years and 4.9% at 4 years (Figure 1A). The patients with HCC (HCC+ group) showed significantly lower levels of platelets and albumin and higher levels of total bilirubin, hyaluronic acid, AFP and the FIB-4 index than those without HCC (HCC− group) (Table 1). The serum TSP-2 levels were significantly higher in the HCC+ group than in the HCC− group (Figure 1B). The AUROC of the serum TSP-2 level for predicting HCC was 0.70 in the derivation cohort (Figure 1C). We then split the patients into two groups based on the cutoff value of the serum TSP-2 level (86.954 ng/µL) determined by the Youden index of the ROC curve. The cumulative HCC rate was significantly higher in the high TSP-2 group (11.4% at 2 years and 14.1% at 4 years) than in the low TSP-2 group (0.9% at 2 years and 3.0% at 4 years) (Figure 1D). These data indicated that serum TSP-2 levels can be used to identify CHC patients who have a high risk of HCC development after HCV elimination by DAA treatment.

### 3.2. Serum TSP-2 Levels Reflect Liver Fibrosis and Inflammation in CHC Patients after HCV Elimination by DAA Treatment

**Figure 2:**
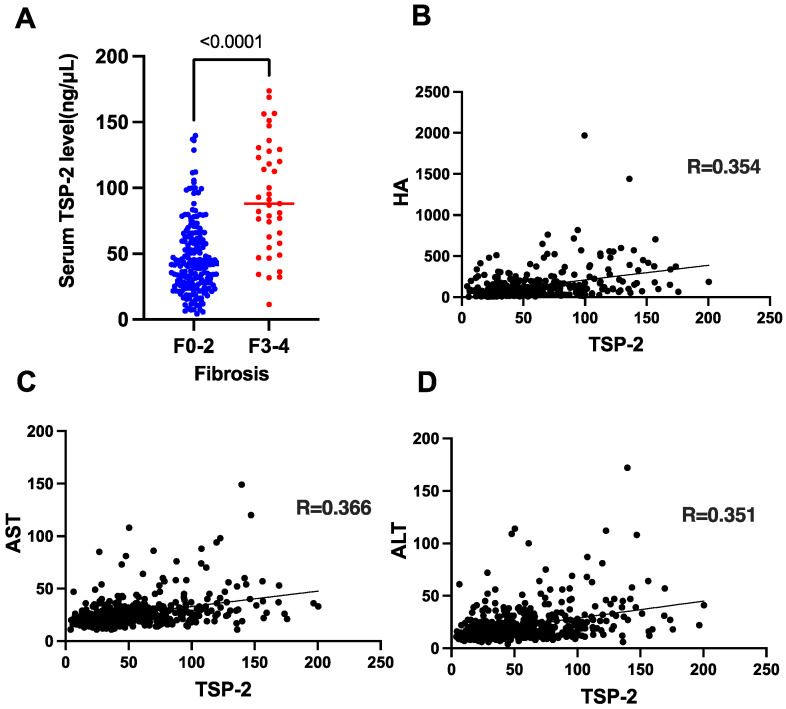
Serum TSP-2 levels reflect liver fibrosis and inflammation in CHC patients after HCV elimination by DAA treatment. (**A**) TSP-2 levels by fibrosis stage in the derivation cohort. (**B**–**D**) Correlation between TSP-2 levels and HA (**B**), AST (**C**), and ALT (**D**) in the derivation cohort.

We next examined the clinical factors associated with serum TSP-2 levels in the derivation cohort. The serum TSP-2 levels were significantly higher in the patients with F3-4 stages than in the patients with F0-2 stages (Figure 2A) and showed a significant positive association with serum hyaluronic acid (HA) levels, a well-known fibrosis marker (Figure 2B, Supplementary Table S1), suggesting that serum TSP-2 levels reflect liver fibrosis. In addition, the serum TSP-2 level was also positively associated with serum AST and ALT levels (Figure 2C,D, Supplementary Table S1), suggesting that serum TSP-2 levels also reflect liver inflammation. These data were consistent with our previous findings that serum TSP-2 levels reflect liver injury and fibrosis in patients with nonalcoholic fatty liver disease (NAFLD) [11].

### 3.3. The AFT Score Composed of AFP, TSP-2 and the FIB-4 Index Stratifies Patients According to the HCC Risk after DAA Treatment

We next sought to identify predictive factors of HCC development in CHC patients after HCV elimination by DAA treatment. In the derivation cohort, univariate analysis using the Cox proportional hazards model identified AFP, the FIB-4 index, and TSP-2 as factors contributing to HCC occurrence (*p* < 0.01) (Table 2). Multivariate analysis showed that all three factors were significant independent predictors of HCC development in this cohort (Table 2). 

**Figure 3:**
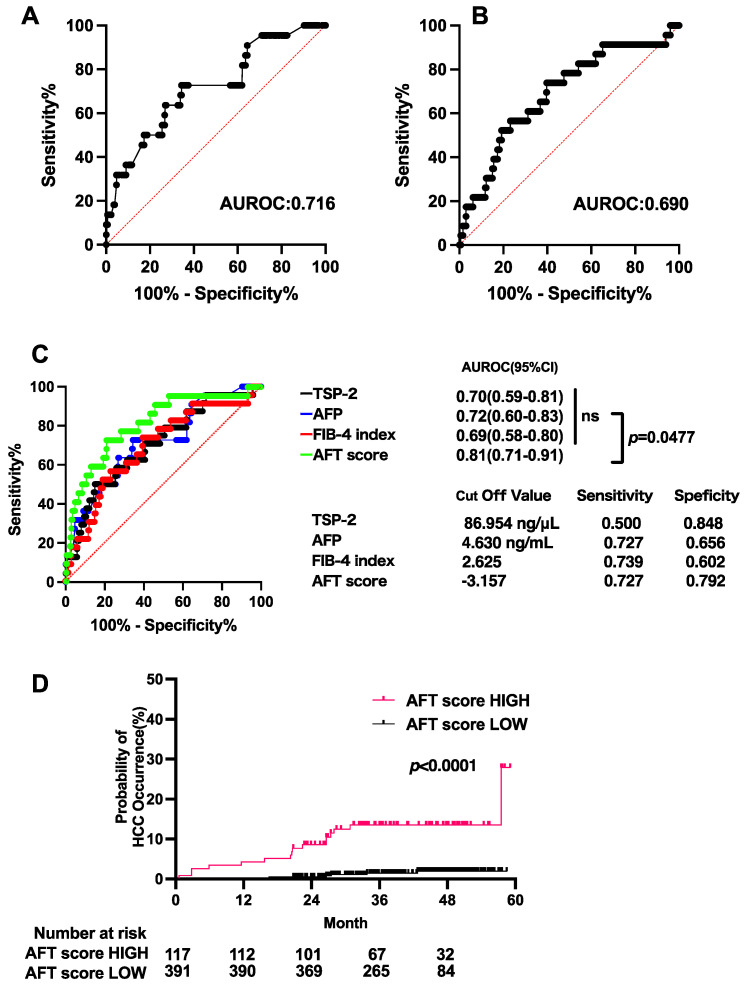
The AFT score composed of AFP, TSP-2 and FIB-4 index stratifies patients according to the HCC risk after DAA treatment. (**A**,**B**) ROC curves of AFP (**A**) and FIB-4 index (**B**) for HCC occurrence in the derivation cohort. (**C**) ROC curves and the AUROCs of AFP, FIB-4 index, TSP-2 and AFT score in the derivation cohort. (**D**) Kaplan–Meier curves for the occurrence of HCC in patients in the derivation cohort stratified by the AFT score.

The AUROCs of AFP and the FIB-4 index to predict HCC occurrence were 0.72 and 0.69, respectively (Figure 3A,B). The predictive power for HCC occurrence was not significantly different among AFP, the FIB-4 index, and TSP-2 (Figure 3C). We thus created a new score (AFT score) composed of these independent predictive factors (AFP, FIB-4 index, and TSP-2) and evaluated its predictive potential. The formula of the AFT score is ‘−5.498859067 + 0.0144285182 × (TSP-2) + 0.2617308791 × (FIB-4 index) + 0.0870554852 × (AFP)’. The AFT score showed an AUROC of 0.81, which was significantly higher than that of each individual factor (Figure 3C). The sensitivity and specificity of the AFT score for predicting HCC occurrence were 0.727 and 0.792, respectively (Figure 3C). The cumulative HCC rate was significantly higher in the high AFT score group (8.6% at 2 years and 13.5% at 4 years) than in the low AFT score group (0.5% at 2 years and 1.9 at 4 years) (Figure 3D). Time-dependent ROC analysis showed that the AUROC value of the AFT score was consistently high for predicting the 1-, 2-, 3-, and 4-year occurrence of HCC (AUROC; 0.801, 0.840, 0.816, and 0.783, respectively) (Supplementary Figure S2). Taken together, the AFT scores stratified patients according to their HCC risk after DAA treatment.

### 3.4. The TSP-2 and AFT Scores Stratified Patients According to HCC Risk in the Validation Cohort

**Figure 4:**
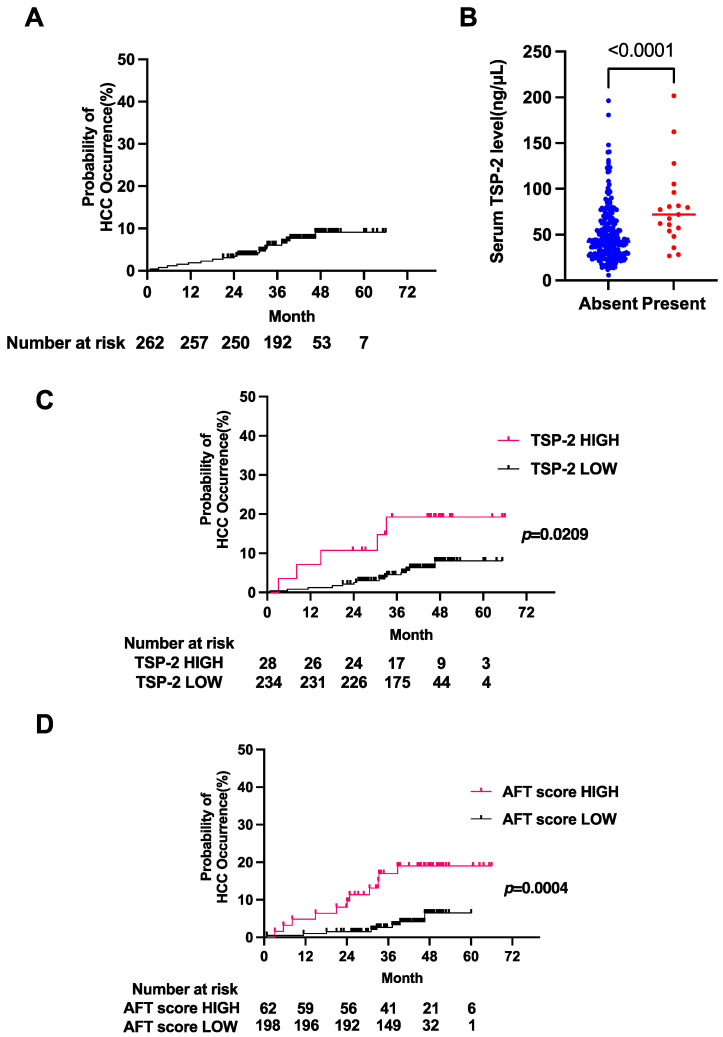
The TSP-2 and AFT scores stratified patients according to HCC risk in the validation cohort. (**A**) Kaplan–Meier curve for HCC occurrence. (**B**) Serum TSP-2 levels in the HCC+ group and HCC− group. (**C**) Kaplan–Meier curves for the occurrence of HCC in patients stratified by serum TSP-2 levels. (**D**) Kaplan–Meier curves for the occurrence of HCC in patients stratified by the AFT score.

We then evaluated the predictive value of AFT score as well as serum TSP-2 level in the validation cohort. In the validation cohort, the median age was 68 years, and the percentage of males was 34.7% (Supplementary Table S2). A total of 19 patients (7.3%) developed HCC during the 43.6-month median observation period, and the cumulative HCC rate after DAA treatment was 3.4% at 2 years and 9.1% at 4 years (Supplementary Table S2, Figure 4A). There was no significant difference in HCC occurrence during the observation period between the derivation and validation cohorts. The patients with HCC occurrence (HCC+ group) showed significantly lower platelet counts, lower levels of WBCs, HCV-RNA, and albumin and higher levels of age, total bilirubin, hyaluronic acid, AFP and the FIB-4 index than those without HCC occurrence (HCC− group) (Supplementary Table S2). The serum TSP-2 levels were significantly higher in the HCC+ group than in the HCC− group (Figure 4B). When we stratified the patients based on the same cutoff value of serum TSP-2 level as determined in the derivation cohort, the cumulative HCC rate was significantly higher in the high TSP-2 group (10.7% at 2 years and 19.3% at 4 years) than in the low TSP-2 group (2.6% at 2 years and 8.1% at 4 years) (Figure 4C). Similarly, the AFT score can also be used to stratify the risk of HCC occurrence after DAA therapy in the validation cohort. The cumulative HCC occurrence rate in the AFT score high group was 9.7% at 2 years and 19.0% at 4 years, while the cumulative HCC occurrence rate in the AFT score low group was 1.5% at 2 years and 6.5% at 4 years (Figure 4D). Taken together, TSP-2 and AFT scores can be used to stratify the patients based on the risk of HCC occurrence in the validation cohort.

### 3.5. Serum TSP-2 Level Identifies Patients with a High Risk of HCC Occurrence among Patients with FIB-4 Index < 3.25

**Figure 5:**
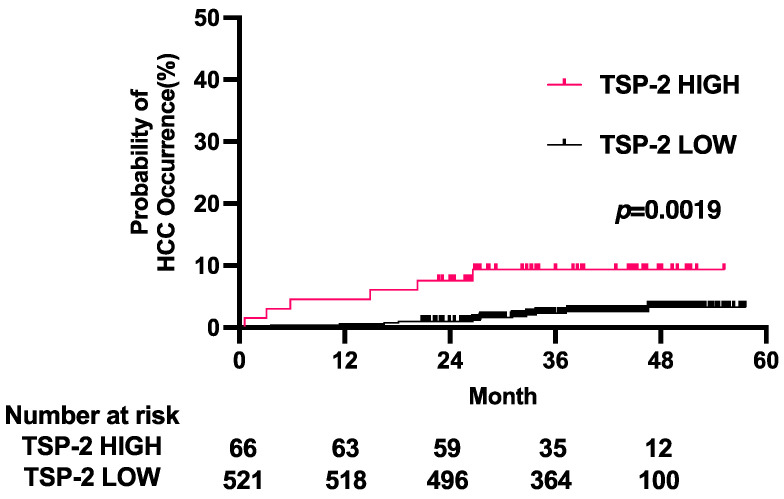
The serum TSP-2 level identifies patients with a high risk of HCC occurrence among patients with a FIB-4 index < 3.25. Kaplan–Meier curves for the occurrence of HCC in patients with a FIB-4 index less than 3.25 among all the cohorts stratified by serum TSP-2 levels.

According to the EASL guidelines, active routine surveillance is not recommended for the group with a FIB-4 index less than 3.25 because their livers are not highly fibrotic and they are at a lower risk of developing HCC in the future [10]. However, even among the 587 patients with a FIB-4 index less than 3.25 in our entire cohort, 19 patients developed HCC during the median 42.4-month observation period, suggesting the importance of developing biomarkers to identify these patients. We thus finally evaluated the potential value of TSP-2 to identify those patients. The cumulative HCC occurrence rate in the high serum TSP-2 group was 7.6% at 2 years and 9.4% at 4 years, while the cumulative HCC occurrence rate in the low serum TSP-2 group was 1.0% at 2 years and 3.3% at 4 years (Figure 5). Collectively, serum TSP-2 levels could identify patients with a FIB-4 index less than 3.25 who may have a high risk of developing HCC.

## 4. Discussion

TSP-2 is a member of the thrombospondin family, which is known to be a matricellular glycoprotein that regulates cell-to-cell and cell-to-matrix interactions [12]. Thrombospondins are known to be involved in tissue repair, angiogenesis, connective tissue organization, and so on [12]. TSP-2 is secreted from the fibrotic liver, and we have recently reported that serum TSP-2 is a novel biomarker for detecting NASH, advanced fibrosis, and predicting HCC occurrence among nonalcoholic fatty liver disease (NAFLD) patients [11]. Similar findings were also reported from two different labs, indicating the validity of TSP-2 as a useful biomarker to determine the activity and fibrosis stage of NAFLD [13,14]. On the other hand, the potential of TSP-2 as a biomarker to predict future HCC occurrence among CHC patients who had HCV eliminated by DAA treatment has never been investigated. In this study, we first examined the serum TSP-2 levels at the end of DAA treatment in 524 CHC patients in the derivation cohort and found that the serum TSP-2 levels were significantly higher in the patients who later developed HCC than in those who did not. We subsequently showed that the serum TSP-2 levels predicted HCC occurrence with an AUROC of 0.7 and thus was used to stratify DAA-treated patients according to the risk of HCC occurrence. Similar results were confirmed in the validation cohort. Moreover, even among the possible noncirrhotic patients selected by a FIB-4 index < 3.25, serum TSP-2 levels were used to stratify patients according to the risk of HCC occurrence. We thus reported here for the first time the usefulness of serum TSP-2 levels in identifying CHC patients who have a high risk of HCC development after HCV elimination by DAA treatment. In the current study, consistent with the results from our previous NAFLD study [11], we found that serum TSP-2 levels were correlated with markers of liver fibrosis and injury, which account for both accumulated and incoming HCC risk, respectively, leading to the high potential to predict HCC occurrence even after HCV eradication.

Several biomarkers available in daily practice have been reported to be able to predict future HCC development after DAA treatment. Liver fibrosis stage is obviously one of the most important predictors for HCC occurrence after DAA treatment [15]. The FIB-4 index, a noninvasive test composed of AST, ALT, platelets and age, can be used to predict the stage of liver fibrosis and has been shown to be a predictive marker of HCC occurrence in patients with a variety of liver diseases [16,17,18,19]. Several groups recently reported that a FIB-4 index higher than 3.25 could be used to identify a high-risk population of CHC patients after SVR secondary to DAA treatment, both in the presence and absence of cirrhosis [20,21,22,23]. Based on these observations, both the EASL and AASLD guidelines recommended HCC surveillance after SVR in patients with a FIB-4 > 3.25 [9,10]. Alpha fetoprotein (AFP) is a well-known diagnostic and prognostic biomarker used clinically for HCC, and a recent study revealed that posttreatment AFP levels could be used to predict HCC occurrence in advanced chronic liver disease from European and Japanese patients who achieved SVR after DAA treatment [20,21,24]. In our current study, consistent with these previous reports, the FIB-4 index and AFP, as well as TSP-2, were also both independent predictors of HCC occurrence. However, the AUROCs of these factors were still approximately 0.7, facilitating the establishment of a new combined scoring system named the AFT score with AFP, FIB-4 index and TSP-2. Our new score predicted HCC occurrence at an AUROC of 0.81, which was significantly higher than that of each individual factor, with good sensitivity (0.727) and specificity (0.792) in the derivation cohort. We also confirmed that the AFT score was capable of discriminating patients with a high risk of developing HCC in the validation cohort. Importantly, the time-dependent ROC analysis showed that the AUROCs of the AFT score predicting HCC occurrence were consistently high throughout the 4-year observation period, indicating the robustness of this predictive biomarker.

Several new serum biomarkers to predict HCC occurrence have also been investigated at the preclinical stage [25]. Nagata H et al. predicted HCC occurrence in CHC patients treated with DAA by measuring the posttreatment levels of serum Wisteria floribunda agglutinin positive Mac-2 binding protein (WFA + M2BP) [26]. WFA + -M2BP is secreted from hepatic stellate cells (HSCs) and promotes their extracellular matrix production via interaction with Mac2-expressing Kupffer cells, possibly contributing to a supportive microenvironment for HCC growth [27]. Although the cohort size was very small, Debes J.D. et al. measured pretreatment serum levels of a variety of immune mediators in CHC patients who underwent DAA therapy, showing that nine cytokines, including MIG, IL22, TRAIL, APRIL, VEGF, IL3, TWEAK, SCF and IL21, could identify the patients who developed de novo HCC with an AUROC > 0.8 [28]. Growth differentiation factor 15 (GDF15) is a cytokine induced by oxidative stress or mitochondrial dysfunction, and we have reported that serum GDF15 predicts HCC occurrence after DAA treatment [29]. Meanwhile, all these markers, including ours, are still in the exploratory phases, and none of these markers have been prospectively validated. Therefore, further accurate prospective evaluations of these biomarkers need to be performed in parallel to select the best candidate for clinical practice.

The limitations of this study are as follows: (1) our research design is a retrospective study, (2) there is no racial diversity, (3) the observation period is still relatively short (a 41.5 months median observation period), (4) information of some potentially important clinical variables is missing (e.g., alcohol consumption and smoking habit) and (5) we only evaluated the utility of serum TSP-2 levels at the end of DAA treatment. Thus, the utility of serum TSP-2 levels long after SVR should be evaluated in the future. In addition, most of the patients underwent only abdominal ultrasonography for HCC surveillance at the enrollment of this study, so we did not exclude the possibility that small HCC already existed especially in patients who developed HCC within 6 months of follow-up. To address this concern, we excluded the patients who developed HCC within 6 months after DAA treatments from the entire cohort. Even in this cohort, serum TSP-2 levels were also significantly higher in patients who had developed HCC than in patients who had not developed HCC (Supplementary Figure S3A). TSP-2, AFP, and the FIB-4 index were independent risk factors for predicting HCC occurrence after DAA treatments. The AUROC of the AFT score for predicting HCC occurrence was higher than that of TSP-2, AFP, and the FIB-4 index (Supplementary Figure S3B). HCC occurrence rate was significantly higher in patients with high TSP-2 levels or high AFT scores than that in patients with low TSP-2 levels or low AFT scores, respectively (Supplementary Figure S3C). Taken together, we believe that TSP-2 and AFT score may be useful for the prediction of de novo HCC occurrence of CHC patients after DAA treatment. 

## 5. Conclusions

Serum TSP-2 levels are a predictive biomarker of HCC occurrence in CHC patients after HCV elimination by DAA treatment. The AFT score using TSP-2, AFP, and the FIB-4 index may identify those who require HCC surveillance.

## Figures and Tables

**Table 1:** Characteristics of patients in the derivation cohort.

Factor	Unit	All (N = 524)	Missing (N)	Non HCC (N = 500)	HCC (N = 24)	*p* Value
Age	Years Old	70 (62–77)	0	70 (61–76)	71 (65–79)	0.171
Sex	Male/Female	204/320	0	192/308	12/12	0.255
HCV Group	1/2/Other	406/117/1	0	386/113/1	20/4/0	0.771
HCV-RNA(Pre)	Log IU/mL	6.2 (5.6–6.5)	2	6.2 (5.6–6.5)	6.2 (5.4–6.6)	0.976
BMI(Pre)	kg/m^2^	22.4 (20.4–24.3)	29	22.4 (20.4–24.3)	22.9 (20.6–26.0)	0.322
WBC	/μL	4700 (3890–5680)	48	4700 (3900–5690)	4290 (3530–5650)	0.409
Hb	g/dL	13.1 (12.0–14.2)	48	13.1 (12.0–14.2)	12.2 (10.7–13.7)	0.071
Plt	×10^4^/μL	16.8 (13.4–20.9)	1	16.9 (13.5–21.0)	13.8 (10.4–20.0)	0.046
AST	U/L	23 (19–28)	1	23 (19–28)	27 (24–34)	0.083
ALT	U/L	17 (12–24)	1	17 (12–24)	20 (15–27)	0.367
T-bil	mg/dL	0.7 (0.6–1.0)	1	0.7 (0.6–1.0)	0.8 (0.6–1.0)	0.030
eGFR	mL/min/1.73 m^2^	68.9 (60–80)	48	69 (60–80)	66.3 (60.9–80.4)	0.742
CRP	mg/dL	0.1 (0.02–0.1)	113	0.1 (0.02–0.1)	0.1 (0.05–0.1)	0.149
HbA1c	%	5.5 (5.2–5.9)	75	5.5 (5.2–5.9)	5.3 (5.0–5.8)	0.725
Alb	g/dL	4.1 (3.9–4.3)	8	4.1 (3.9–4.3)	4.0 (3.7–4.2)	0.011
Hyaluronic Acid	ng/mL	84 (39–163)	184	81 (38–156)	149 (103–339)	0.031
AFP	ng/mL	4.0 (2.7–5.9)	16	4.0 (2.6–5.5)	6.0 (3.3–10)	<0.0001
DCP	mAU/mL	18 (14–21)	160	18 (14–21)	16 (11–20)	0.386
FIB-4 index		2.3 (1.7–3.2)	1	2.3 (1.6–3.2)	3.5 (2.4–4.3)	0.0007
TSP-2	ng/μL	44.9 (29.0–72.4)	0	44.2 (28.3–71.2)	78.2 (44.1–111)	<0.0001

Abbreviations: HCC, hepatocellular carcinoma; HCV, hepatitis C virus; RNA, ribonucleic acid; BMI, body mass index; WBC, white blood cell; Hb, hemoglobin; Plt, platelet; AST, aspartate aminotransferase; ALT, alanine aminotransferase; T-bil, total bilirubin; eGFR, estimated glomerular filtration rate; CRP, c-reactive protein; HbA1c, hemoglobin A1c; Alb, albumin; AFP, α-fetoprotein; DCP, des-γ-carboxy prothrombin; FIB-4, fibrosis-4; TSP-2, thrombospondin-2.

**Table 2:** Univariate and multivariate analysis in the derivation cohort.

Factor	Unit	Univariate Analysis *p* Value	Multivariate Analysis *p* Value
Age	Years Old	0.175	
Sex		0.244	
HCV Group		0.700	
HCV-RNA(Pre)	Log IU/mL	0.981	
BMI(Pre)	kg/m^2^	0.369	
WBC	/μL	0.412	
Hb	g/dL	0.078	
Plt	×10^4^/μL	0.056	
AST	U/L	0.106	
ALT	U/L	0.343	
T-bil	mg/dL	0.042	
eGFR	mL/min/1.73 m^2^	0.723	
CRP	mg/dL	0.228	
HbA1c	%	0.740	
Alb	g/dL	0.011	
Hyaluronic Acid	ng/mL	0.109	
AFP	ng/mL	<0.0001	<0.0001
DCP	mAU/mL	0.399	
FIB-4 index		0.006	0.015
TSP-2	ng/μL	0.0002	0.003

Abbreviations: HCV, hepatitis C virus; RNA, ribonucleic acid; BMI, body mass index; WBC, white blood cell; Hb, hemoglobin; Plt, platelet; AST, aspartate aminotransferase; ALT, alanine aminotransferase; T-bil, total bilirubin; eGFR, estimated glomerular filtration rate; CRP, c-reactive protein; HbA1c, hemoglobin A1c; Alb, albumin; AFP, α-fetoprotein; DCP, des-γ-carboxy prothrombin; FIB-4, fibrosis-4, TSP-2, thrombospondin-2.

## Data Availability

The data presented in this study are available on request from the corresponding author.

## Supplementary Materials

The following supporting information can be downloaded at: https://www.mdpi.com/article/10.3390/cancers15020463/s1, Figure S1: 786 patients who fulfilled the following criteria from our multicenter prospective observational cohort: those with SVR achievement, those with complete medical records, those without a past HCC history, and those who provided agreement for serum storage, Figure S2: Time-dependent ROC of the occurrence of HCC 1-, 2-, 3-, and 4-year after DAA treatment, Figure S3: Analysis of the cohort without patients who developed HCC within 6 months of follow-up, Table S1: Correlation coefficient between TSP-2 and other clinical variables in the derivation cohort, Table S2: Characteristics of patients in the validation cohort.
