# Supplementary material for: Thrombospondin-2 as a Predictive Biomarker for Hepatocellular Carcinoma after Hepatitis C Virus Elimination by Direct-Acting Antiviral
Source: Cancers (Basel). 2023 Jan 11;15(2):463. doi: 10.3390/cancers15020463 (PMC9856394; doi:10.3390/cancers15020463)
Supplement: Supplementary file 1 [file cancers-15-00463-s001.zip › cancers-2107283-supplementary.pdf]

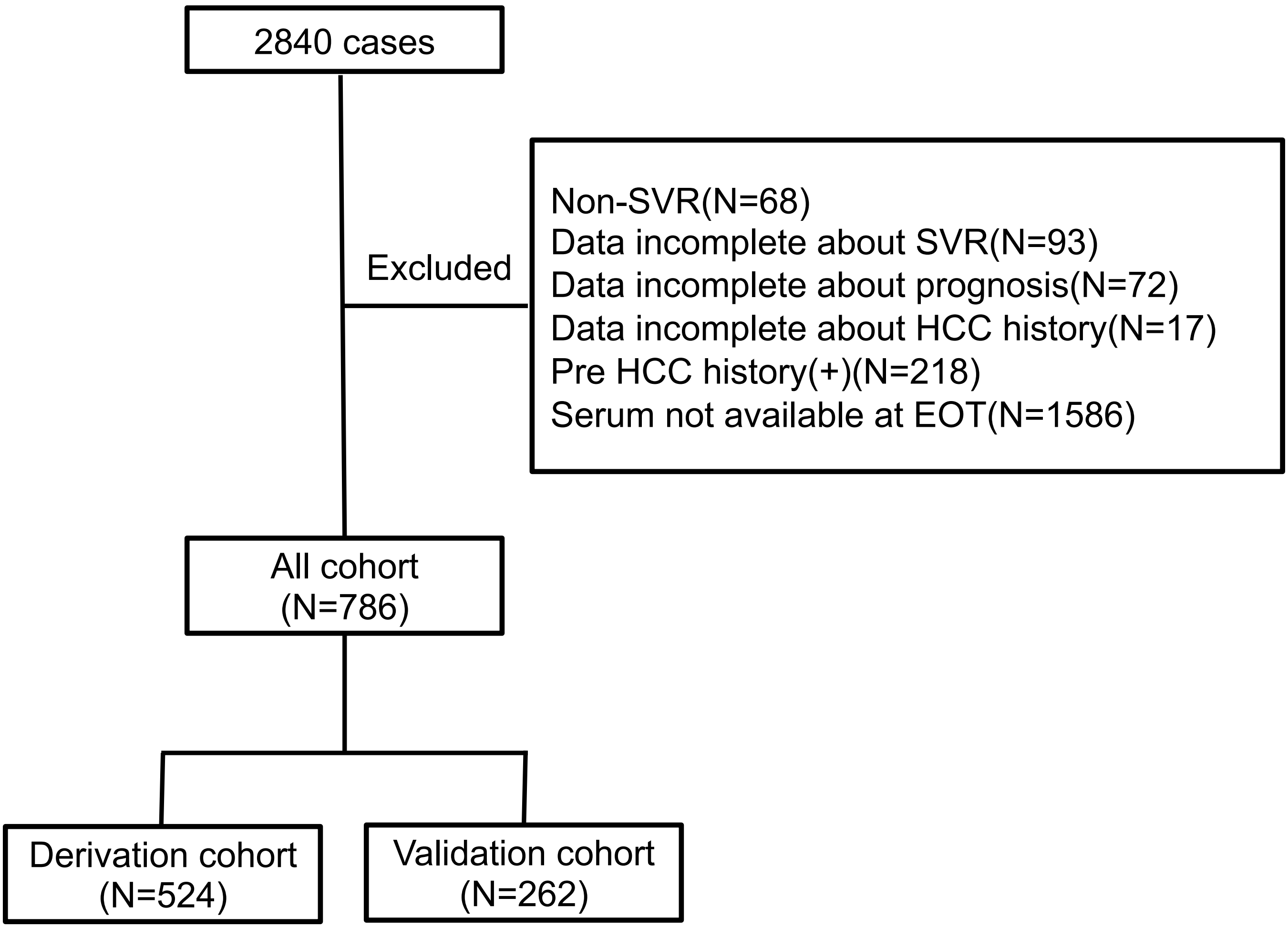

Supplementary Figure S1

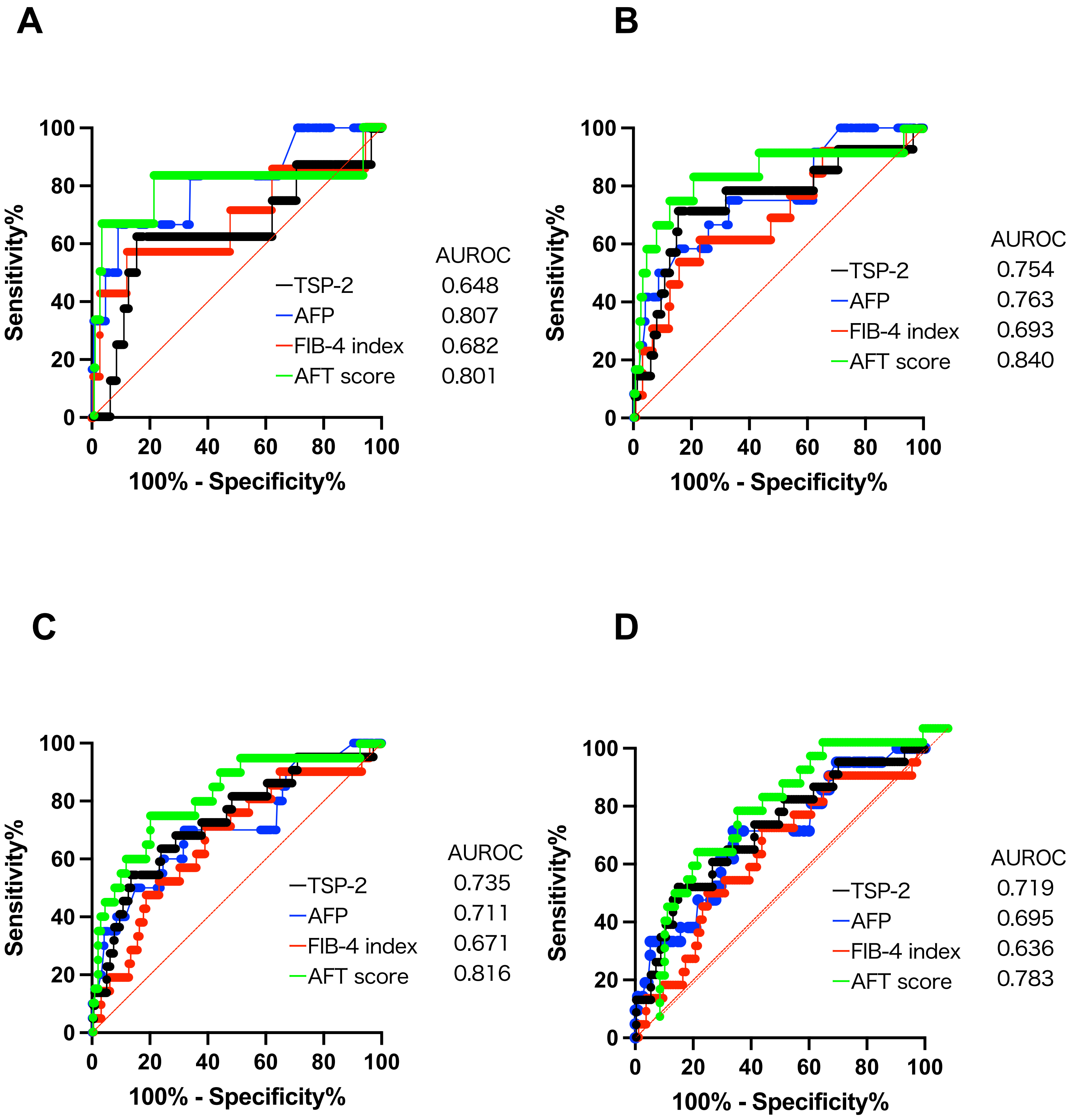

Supplementary Figure S2

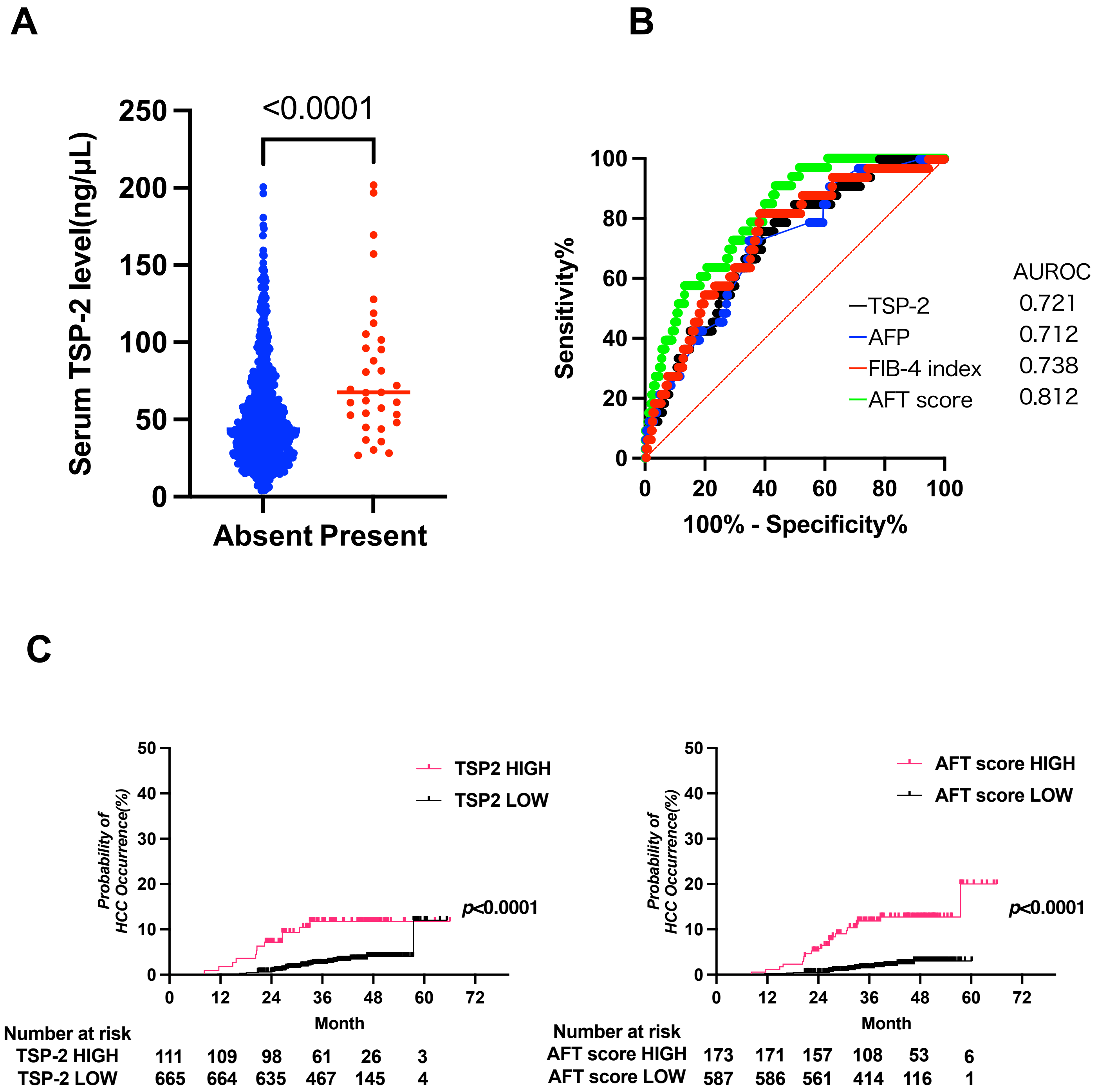

Supplementary Figure S3

Supple Table S1. Correlation coefficient between TSP-2 and another factor in the derivation cohort

Factor	R	*p* value
AST	0.366	<0.0001
Hyaluronic Acid	0.354	<0.0001
ALT	0.351	<0.0001
Plt	-0.247	<0.0001
Alb	-0.150	0.0006
HbA1c	0.116	0.014
AFP	0.103	0.021
BMI	0.074	0.102
Hb	0.067	0.142
DCP	0.041	0.433
T-Bil	-0.041	0.302
Age	-0.023	0.593

Abbreviations: AST,aspartate aminotransferase; ALT,alanine aminotransferase; FIB-4,fibrosis-4
Plt,platelet; Alb,albumin; HbA1c,hemoglobin A1c; AFP,α-fetoprotein
Hb,hemoglobin; DCP,des-γ-carboxy prothrombin; T-bil,total bilirubin

Supplementary Table S2. Characteristics of patients in the validation cohort

		All(N=262)	Missing(N)	Non HCC(N=243)	HCC(N=19)	*p* value
Age	Years Old	68(58-74)	0	67(57-74)	75(68-77)	0.001
Sex	Male/Female	91/171	0	86/157	5/14	0.424
HCV Group	1/2/Other	224/29/0	9	215/28	9/1	0.882
HCV-RNA(Pre)	Log IU/mL	6.2(5.7-6.5)	9	6.2(5.7-6.5)	5.7(4.7-5.9)	0.005
BMI(Pre)	kg/m^2	22.6(20.1-24.7)	0	22.6(20.0-24.6)	23.3(20.3-26.9)	0.415
WBC	/μL	4720(4080-6060)	2	4910(4140-6140)	3850(3320-4620)	0.005
Hb	g/dL	13.3(12.3-14.3)	2	13.4(12.4-14.4)	12.8(11.6-13.7)	0.127
Plt	$\times 10^4/\mu L$	16.7(12.8-21.7)	1	16.9(13.2-22.1)	12.9(10.5-17.8)	0.028
AST	U/L	24(19-30)	1	23(19-29)	28(22-37)	0.299
ALT	U/L	17(13-26)	1	17(13-26)	20(15-25)	0.637
T-bil	mg/dL	0.7(0.6-0.9)	10	0.7(0.6-0.9)	0.9(0.6-1.7)	0.041
eGFR	$mL/min/1.73m^2$	70.5(61.3-80.7)	10	70.5(61.3-80.6)	71.7(63.0-82.4)	0.838
CRP	mg/dL	0.04(0.04-0.04)	15	0.04(0.04-0.04)	0.05(0.04-0.10)	0.418
HbA1c	%	5.5(5.3-5.9)	12	5.5(5.3-5.9)	5.6(5.0-5.8)	0.619
Alb	g/dL	4.1(3.9-4.3)	1	4.1(3.9-4.3)	3.8(3.6-4.0)	0.0005
Hyaluronic Acid	ng/mL	83(48-165)	13	80(45-157)	371(179-469)	0.005
AFP	ng/mL	4.0(2.8-6.0)	2	4.0(2.6-6.0)	6.0(4.7-9.0)	<0.0001
DCP	mAU/mL	19(15-24)	9	19(15-24)	20(15-27)	0.140
FIB-4 index		2.2(1.5-3.3)	1	2.1(1.5-3.1)	4.0(2.6-5.7)	<0.0001
TSP-2	ng/μL	43.6(29.0-65.2)	0	42.4(28.9-62.1)	72.1(54.0-96.2)	<0.0001

Abbreviations: HCC,hepatocellular carcinoma; HCV,hepatitis C virus; RNA,ribonucleic acid; BMI, body mass index; WBC,white blood cell; Hb,hemoglobin
Plt,platelet; AST,aspartate aminotransferase; ALT,alanine aminotransferase; T-bil,total bilirubin; eGFR,estimated glomerular filtration rate
CRP,c-reactive protein; HbA1c,hemoglobin A1c; Alb,albumin; AFP,α-fetoprotein; DCP,des-γ-carboxy prothrombin
FIB-4,fibrosis-4; TSP-2,thrombospondin-2
